# COVID-19 global pandemic planning: Presence of SARS-CoV-2 fomites in a university hospital setting

**DOI:** 10.1177/15353702211024597

**Published:** 2021-07-04

**Authors:** Christopher Bartlett, Jens Langsjoen, Qiuying Cheng, Alexandra V Yingling, Myissa Weiss, Steven Bradfute, Douglas J Perkins, Ivy Hurwitz

**Affiliations:** 1 Department of Internal Medicine, University of New Mexico Health Sciences Center, Albuquerque, NM 87131, USA; 2 School of Medicine, University of New Mexico Health Sciences Center, Albuquerque, NM 87131, USA

**Keywords:** Coronavirus, SARS-CoV-2, fomites

## Abstract

As severe acute respiratory syndrome coronavirus 2 (SARS-CoV-2) has surged across the globe, great effort has been expended to understand mechanisms of transmission and spread. From a hospital perspective, this topic is critical to limit and prevent SARS-CoV-2 iatrogenic transmission within the healthcare environment. Currently, the virus is believed to be transmitted primarily through respiratory droplets, but a growing body of evidence suggests that spread is also possible through aerosolized particles and fomites. Amidst a growing volume of patients with coronavirus disease 2019 (COVID-19), the purpose of this study was to evaluate the potential for SARS-CoV-2 transmission through fomites. Samples collected from the exposed skin of clinicians (n = 42) and high-touch surfaces (n = 40) were collected before and after encounters with COVID-19 patients. Samples were analyzed using two assays: the CDC 2019-nCoV Real-Time Reverse Transcription polymerase chain reaction (RT-qPCR) assay, and a SYBR Green assay that targeted a 121 bp region within the *S*-gene of SARS-CoV-2. None of the samples tested positive with the CDC assay, while two high-touch surface areas tested positive for SARS-CoV-2 using the Spike assay. However, viral culture did not reveal viable SARS-CoV-2 from the positive samples. Overall, the results from this study suggest that SARS-CoV-2 RNA were not widely present either on exposed skin flora or high-touch surface areas in the hospital locations tested. The inability to recover viable virus from samples that tested positive by the molecular assays, however, does not rule out the possibility of SARS-CoV-2 transmission through fomites.

## Impact statement

While the primary route for SARS-CoV-2 transmission occurs through direct inhalation of respiratory droplets, contamination of high-touch surfaces by the virus has the potential to cause indirect nosocomial spread. We tested samples collected from high-touch surface areas outside of COVID-19 patient rooms and on healthcare provider workstations for the presence of SARS-CoV-2 using two PCR-based assays. A SYBR Green assay developed in the laboratory detected the presence of SARS-CoV-2 RNA in two of the collected samples. However, neither of the samples grew viable virus when cultured onto Vero E6 cells. The extensive use of virucidal cleaners on high-touch surfaces likely contributes to the observed results. While our study demonstrated the absence of viable SARS-CoV-2 on sampled locations, the persistence of SARS-CoV-2 on fomites remains undetermined. As such, it is critical that vigilance be exercised to prevent potential viral transmission.

## Introduction

As of 22 April 2021, the coronavirus disease 2019 (COVID-19) pandemic has affected more than 144 million people worldwide.^1^ One route of transmission of severe acute respiratory syndrome coronavirus 2 (SARS-CoV-2), the causative agent of COVID-19, is through inhalation of respiratory droplets and aerosols expelled from an infected individual during coughing/sneezing, talking or exhaling.^2^ While aerosolized particles persist in the air for minutes to hours, exhaled droplets will quickly settle on nearby inanimate objects and surfaces. Touching of these contaminated surfaces, or fomites, by an unsuspecting host can result in self-inoculation of mucous membranes of the mouth, nose, or eyes. In the hospital setting, SARS-CoV-2 contamination has been detected on numerous high-contact surfaces, specifically on bed rails, tables, call panels, and door handles of rooms housing COVID-19 patients.^3–5^ While fomite spread had been associated with nosocomial transmission of other viruses and bacteria,^6,7^ the transfer efficiency of SARS-CoV-2 from fomites to humans remains largely uncharacterized. This study, conducted during the early stages of the COVID-19 pandemic, was designed to evaluate the potential for SARS-CoV-2 transmission through fomites in COVID-19 units at the University of New Mexico Hospital (UNMH). We further investigated whether aerosolized particles from hospitalized COVID-19 patients can potentially contaminate exposed skin surfaces of healthcare providers (HCP) during routine care.

## Materials and methods

*Setting:* UNMH is a 618-bed tertiary care facility serving New Mexico and the surrounding regions. Between 16 April and 30 April 2020, a total of 30 PCR-confirmed non-ICU COVID-19 patients were admitted to UNMH. To prevent potential of transmission of SARS-CoV-2, patients were individually housed in negative pressure isolation rooms that were retrofitted with portable high-efficiency particulate air (HEPA) exhaust fans to provide a minimum of 12 air changes per hour, and at least 2.5 Pa of negative pressure to the adjacent hallway.^8^

*Study participants:* Participants in the study were HCP who were directly caring for non-ICU patients infected with SARS-CoV-2. Exposure was defined as the first clinical encounter of the day between the HCP and a COVID-19 patient who was between 1 and 3 days of hospitalization. Sample collection was completed over a 2-week period between 17 April and 30 April 2020. This study was approved by the UNM Health Sciences Human Research Protections Program (Protocol ID: 20–2180). Written consent was obtained from all participants.

*Fomite sample collection:* Fomite samples were collected with a 2″ × 2″ piece of sterile Whatman paper, pre-soaked with phosphate buffered saline (PBS). To maximize the surface area covered, swabs were collected following an “S” shape pattern. Following collection, swabs were immediately deposited into 50-mL conical tubes containing 5 mL of viral transport medium (VTM). The exterior of the tubes were wiped with Oxivir® disinfectant wipes, placed into a zip-lock bag, and stored at 4°C (2–4 h) until processing.

*Sample collection from HCP:* Fomite samples were collected from participating HCP before and after patient encounters. Pre-exposure samples were collected prior to donning of PPE, while post-exposure samples were collected following PPE doffing and upon exiting the patient’s room. Samples were collected with gloved hands from skin around the nose and mouth. Samples were also collected from the HCP’s exposed skin at the temples, cheeks, and neck. Additional samples were taken from the sides of the HCP’s footwear. The length of each encounter was documented, and participating HCPs completed a brief questionnaire following each encounter (Supplemental Questionnaire).

*Sample collection from high-touch surface areas:* Fomite samples were collected from high-touch surface areas outside the rooms of COVID-19 patients (i.e. donning/doffing stations, doorknobs, door thresholds, and shared workstations (mouse and keyboard)) before and after encounters between the patients and HCP. In addition, fomite samples were collected from high-touch surface areas (i.e. door handles and shared workstations) in the emergency room and other COVID-19 wards in the UNMH.

*Processing of VTM and isolation of viral RNA:* The 50 mL conical tubes, containing the fomite samples collected on Whatman paper in 5 mL of VTM, were centrifuged at 1200 x *g* for 10 min. VTM was aliquoted into three microfuge tubes and stored at –80°C until potential use in viral growth assays (see below). One aliquot was inactivated with an equal volume of 2X DNA/RNA Shield (Zymo Research, Irvine, CA, USA) and stored at –80°C until batch processing for detection of SARS-CoV-2 by Reverse Transcription polymerase chain reaction (RT-qPCR). Isolation of viral RNA was performed on inactivated VTM using the Quick-Viral RNA kit (Zymo Research, Irvine, CA, USA) per manufacturer’s protocol. Viral RNA was eluted with 50 µL of DNase/RNase-free water.

*Detection of SARS-CoV-2 by RT-qPCR:* The presence of SARS-CoV-2 in the samples was determined using two different PCR-based assays. Primers and probes (N1, N2, and RP) from the CDC 2019 Real-time RT-qPCR diagnostic panel were initially used. Positive controls used in these reactions included SARS-CoV-2 genomic RNA isolated from isolate USA-WA1/2020 (BEI Resources, Manassas, VA, USA), as well as the 2019-nCoV-N and Hs-RPP30 control plasmids (IDT, Coralville, IA, USA). RT-qPCR was performed per manufacturer’s protocol using TaqPath 1-step RT-qPCR master mix (ThermoFisher Scientific, Waltham, MA, USA).

Samples were also analyzed using a second SYBR Green-based assay that specifically targeted a 121 bp region within the *S*-gene of SARS-CoV-2. This Spike assay utilized primers RBD-qF1, 5′-CAATGGTTTAACAGGCACAGG-3′, and RBD-qR1, 5′-CTCGTGTGTCTGTGGTCCG-3′, as described.^9^ SARS-CoV-2 RNA and a plasmid harboring a fragment of the *S*-gene (position 1629–1749) were used as controls in these reactions. Each 20 µL reaction consisted of 5 µL of 4× TaqPath 1-step RT-qPCR master mix (ThermoFisher Scientific, Waltham, MA, USA), 0.4 µL of 50× ROX reference dye (Lumiprobe, Hunt Valley, MD, USA), 0.2 µL of 100× dsGreen (Lumiprobe, Hunt Valley, MD, USA), 0.4 µL of each primer (IDT, Coralville, IA, USA; working stock 10 µM) and 5 µL of template RNA.

Reverse transcription and amplification conditions for both RT-qPCR assays were performed at 25°C for 2 min, 50°C for 15 min, 95°C for 2 min, followed by 45 cycles of 95°C for 3 s and 55°C for 30 s. The default melting curve step was included following the final amplification cycle for the Spike assay. All RT-qPCR reactions were performed on the ABI StepOnePlus Real-Time PCR system (ThermoFisher Scientific, Waltham, MA, USA).

*Viral cultures:* SARS-CoV-2 viral cultures were conducted in a biosafety level 3 laboratory at UNM Health Science Center (UNM HSC) with approved protocols. Briefly, Vero E6 cells were grown to 90% confluency in a 24-well plate with Dulbecco’s minimal essential medium (DMEM) supplemented with 10% fetal calf serum (FCS) and 1% penicillin/streptomycin. Selected VTM samples that tested positive with the RT-qPCR, along with a selection of samples that tested negative were thawed and filtered to ensure sterility (0.22 µm filter). Each well of Vero E6 cells were inoculated with 100 µL of filtered VTM. Cells were incubated at 37°C in a humidified incubator for 8 days. Cultures were monitored every 48 h for cytopathic effect (CPE) by microscopy. Control conditions were performed with either the SARS-CoV-2 isolate USA-WA1/2020 (BEI Resources, Manassas, VA, USA) or with media only. Infectious SARS-CoV-2 was confirmed when CPE was detected in the inoculated wells.

## Results

*Sample collection:* A total of 82 samples were collected during the 2-week study period. Seventy pre- and post-exposure samples were collected from 12 distinct clinical encounters (Tables 1 and 2). Additionally, 12 high-touch surface areas from heavy traffic COVID-19 hospital care areas were collected (Table 3).

**Table 1.** Healthcare personnel fomite samples before and after patient encounters.

		Before encounter					After encounter				
		CDC assay			Spike assay		CDC assay			Spike assay	
Sample type	Encounter	N1 (C_T_)	N2 (C_T_)	RP (C_T_)	C_T_	T_M_	N1 (C_T_)	N2 (C_T_)	RP (C_T_)	C_T_	T_M_
Skin around nose and mouth	Encounter 1	UD	UD	36.0	34.6	74.2	UD	UD	35.9	35.4	73.7
	Encounter 2	UD	UD	33.4	34.0	74.0	UD	UD	31.3	34.0	74.0
	Encounter 3	UD	UD	31.3	34.2	74.3	UD	UD	32.0	34.5	74.3
	Encounter 4	UD	UD	33.4	38.3	73.4	UD	UD	33.1	34.6	74.2
	Encounter 5	UD	UD	33.8	34.1	74.6	UD	UD	32.7	35.6	74.2
	Encounter 6	UD	UD	34.9	36.9	74.4	UD	UD	35.1	34.7	74.6
	Encounter 7	UD	UD	33.0	36.5	76.0	UD	UD	34.1	35.1	75.8
	Encounter 8	–	–	–	–	–	UD	UD	UD	37.7	74.0
	Encounter 9	–	–	–	–	–	UD	UD	36.1	36.2	74.9
	Encounter 10	UD	UD	38.0	35.9	74.2	UD	UD	35.4	37.1	76.7
	Encounter 11	–	–	–	–	–	UD	UD	35.6	37.7	74.0
	Encounter 12	UD	UD	33.9	37.4	74.0	UD	UD	34.5	36.7	74.5
Exposed skin at temples, cheek, and neck	Encounter 1	UD	UD	37.0	33.6	74.8	UD	UD	37.9	34.1	74.3
	Encounter 2	UD	UD	35.8	34.1	74.2	UD	UD	35.2	34.5	74.3
	Encounter 3	UD	UD	35.2	33.3	75.7	UD	UD	36.7	33.5	74.6
	Encounter 4	UD	UD	33.4	38.3	73.4	UD	UD	33.1	34.6	74.6
	Encounter 5	UD	UD	34.4	34.2	74.3	UD	UD	34.9	34.5	74.2
	Encounter 6	UD	UD	34.2	36.0	74.0	UD	UD	32.9	34.5	74.3
	Encounter 7	UD	UD	32.7	33.5	75.8	UD	UD	32.9	34.0	76.5
	Encounter 8	–	–	–	–	–	UD	UD	37.3	38.5	74.3
	Encounter 9	–	–	–	–	–	UD	UD	35.4	35.6	75.1
	Encounter 10	UD	UD	36.9	36.5	76.6	UD	UD	38.0	37.6	74.0
	Encounter 11	–	–	–	–	–	UD	UD	35.7	37.1	74.0
	Encounter 12	UD	UD	37.2	37.3	73.1	UD	UD	39.1	37.2	73.6
Sides of footwear	Encounter 5	UD	UD	36.6	34.1	74.2	UD	UD	35.2	35.7	74.1
	Encounter 6	UD	UD	36.0	33.8	74.6	UD	UD	35.3	35.3	78.1
	Encounter 7	UD	UD	UD	37.0	72.2	UD	UD	37.9	37.5	74.9
	Encounter 10	UD	UD	35.2	37.0	77.0	UD	UD	39.0	38.3	74.8
	Encounter 12	UD	UD	UD	37.2	73.7	UD	UD	39.9	38.0	74.0
SARS-CoV-2	10^4^ copies	23.2	24.9	–	20.8	79.8	23.2	24.9	–	20.8	79.8
	10^3^ copies	28.5	31.3	–	26.5	79.8	28.5	31.3	–	26.5	79.8
	10^2^ copies	32.8	36.2	–	32.7	79.1	32.8	36.2	–	32.7	79.1

N1 and N2: CDC 2019-nCoV primer and probe mixes that target two viral nucleocapsid (N) genes for specific detection of SARS-CoV-2.

RP: primer and probe set that targets human RNase P gene; C_T_: cycle threshold; T_M_: melting temperature; UD: undetermined.

**Table 2. table2-15353702211024597:** High touch environmental fomite samples outside patient rooms before and after encounters.

Sample type	Encounter	Before encounter					After encounter				
		CDC assay			Spike assay		CDC assay			Spike assay	
		N1 (C_T_)	N2 (C_T_)	RP (C_T_)	C_T_	T_M_	N1 (C_T_)	N2 (C_T_)	RP (C_T_)	C_T_	T_M_
Donning/doffing stations, doorknobs, thresholds, and shared workstations	Encounter 1	UD	UD	35.0	34.9	75.0	UD	UD	UD	*30.6*	*80.5*
	Encounter 2	UD	UD	UD	35.0	74.3	UD	UD	UD	34.6	74.4
	Encounter 3	UD	UD	UD	33.9	74.2	UD	UD	UD	34.9	74.4
	Encounter 4	UD	UD	UD	34.2	74.0	UD	UD	UD	35.5	74.4
	Encounter 5	UD	UD	38.3	34.2	74.9	UD	UD	36.5	34.3	74.3
	Encounter 6	UD	UD	UD	34.5	74.1	UD	UD	35.7	34.9	74.3
	Encounter 7	UD	UD	UD	37.0	73.8	UD	UD	36.7	36.9	74.3
	Encounter 10	UD	UD	38.9	37.4	74.2	UD	UD	35.8	36.8	73.7
	Encounter 12	UD	UD	UD	36.0	73.9	UD	UD	36.2	38.1	74.2
SARS-CoV-2	10^4^ copies	23.6	25.2	–	21.3	79.8	23.6	25.2	–	21.3	79.8
	10^3^ copies	27.4	29.7	–	27.1	79.6	27.4	29.7	–	27.1	79.6
	10^2^ copies	30.8	31.4	–	32.3	79.4	30.8	31.4	–	32.3	79.4

N1 and N2: CDC 2019-nCoV primer and probe mixes that target two viral nucleocapsid (N) genes for specific detection of SARS-CoV-2.

RP: Primer and probe set that targets human RNase P gene; C_T_: Cycle threshold; T_M_: melting temperature; UD: undetermined.

**Table 3.** High touch environmental fomite samples.

Sample type	Location	CDC assay			Spike assay	
		N1 (C_T_)	N2 (C_T_)	RP (C_T_)	C_T_	T_M_
Door handle	Entry push button (Emergency Department)	UD	UD	UD	38.2	72.5
	Exit push button (Emergency Department)	UD	UD	39.0	36.9	76.1
	Physician workroom (Emergency Department)	UD	UD	36.1	***37.8***	***80.2***
	Locker room (Emergency Department)	UD	UD	37.3	37.1	74.6
	Family consultation room (Emergency Department)	UD	UD	UD	37.3	74.2
	Resident workroom (Internal Medicine)	UD	UD	UD	37.4	74.5
	Nurses breakroom (Internal Medicine)	UD	UD	40.8	36.8	75.8
	Library workroom (Internal Medicine)	UD	UD	37.8	36.0	75.2
	Stairwell (Internal Medicine)	UD	UD	36.9	36.0	75.2
	Hospital Medicine office (Hospital Medicine)	UD	UD	38.8	34.5	76.1
**Other**	Respiratory Care Center workstation(Emergency Department)^a^	UD	UD	37.4	36.5	74.5
	Nursing workstation (Internal Medicine)^a^	UD	UD	39.4	38.5	74.2
**SARS-** **CoV-2**	10^4^ copies	23.4	25.2	–	23.4	79.6
	10^3^ copies	28.9	32.9	–	28.9	79.6
	10^2^ copies	32.8	36.9	–	33.7	79.1

^a^Indicates samples collected from mouse and keyboards at shared workstations.

N1 and N2: CDC 2019-nCoV primer and probe mixes that target two viral nucleocapsid (N) genes for specific detection of SARS-CoV-2.

RP: primer and probe set that targets human RNase P gene; C_T_: cycle threshold; T_m_: melting temperature;

UD: undetermined.

*Clinical encounters:* In all clinical encounters, the HCP wore a hair bouffant, surgical mask, contact gown, and gloves. In four cases, an N95 respirator was worn under the surgical mask. Safety glasses were worn on 11 encounters, while a face shield was worn by the HCP on 1 encounter.

The HCP had direct patient contact in 8 of the 12 clinical encounters, which occurred when a physical exam was performed. Patients coughed during 6 clinical encounters, spoke during 11, wore a surgical mask in 5, and were wearing a nasal cannula in 4 of the encounters. One HCP touched their exposed skin during the clinical encounter. The average time for the clinical encounters was 8.2 min: (range: 2–20 min).

*Detection of viral RNA using the CDC RT-qPCR panel:* The CDC RT-PCR diagnostic panel (N1 and N2) did not detect any viral RNA on the skin or footwear of the HCP either before or after the patient encounters, despite detection of RNAse P (RP) in nearly all samples (Table 1). In addition, the N1 and N2 assays failed to detect viral RNA on high-touch surface areas outside the patient rooms either before or after patient encounters (Table 2). Similarly, the assay did not detect any SARS-CoV-2 viral fomites on the 12 high-touch environmental surfaces that were tested (Table 3).

**Figure 1.**
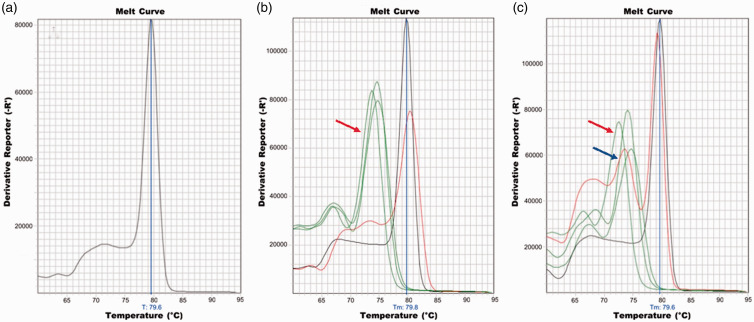
Melting curve analysis of SARS-CoV-2 S-gene (position 1629–1749) amplicon from (a) control SARS-CoV-2 RNA. Plots (b) and (c) show melting curves of various samples from two Spike qPCR assays. In both plots, the control (SARS-CoV-2 S-gene) is shown in black. Putative SARS-CoV-2 contaminated samples are plotted in red. Fomite sample collected from door threshold outside of patient’s room following HCP encounter is shown in plot (b) while fomite collected from a door handle to a physician’s workroom is in plot (c). The green colored peaks (indicated by the red arrows) on plots (b) and (c) are melting curve for non-specific amplicons from other fomite samples. The melting curve of the sample collected from the door handle to the physician’s workroom (plot (c), indicated by the blue arrow) has multiple peaks, suggesting the presence of multiple amplicons.

*Validation of the Spike RT-qPCR assay:* Next, we validated the use of the Spike assay for detection of viral RNA on fomite samples. The efficiency of the Spike assay was evaluated using 10-fold serial dilutions of SARS-CoV-2 RNA (2 to 2 × 10^7^ copies). Each 10-fold dilution corresponded to an increased C_T_ value by an average of 3.8. The measured sensitivity, corresponding to the y-intercept of one copy of viral RNA, corresponded to a C_T_ of 41.76 (Supplemental Figure). The efficiency of the reaction was 82.45%. Melt-curve analysis of the *S*-gene amplicon from SARS-CoV-2 RNA revealed an average melting temperature (T_M_) of 79.6°C (Figure 1(a)).

*Detection of viral RNA using the Spike RT-qPCR assay:* Following validation, the Spike RT-qPCR platform was used to measure SARS-CoV-2 RNA from all fomite samples collected. C_T_ values for the fomite samples ranged from 30.6 to 38.5 (Tables 1 [Table table2-15353702211024597]to 3). However, SYBR Green dye dissociation assays on each amplified sample revealed that only two amplicons had the expected T_M_ of 79.6°C ± 1°C. One sample (C_T_ = 30.6 and T_M_ = 80.5°C) was collected outside the patient’s room on the door threshold following the encounter with the HCP (Encounter 1, Table 2 and Figure 1(b)). Repeated assays on this sample (n = 3) continued to suggest the presence of approximately 1000 copies of viral RNA at this location. The second positive sample (C_T_ = 37.8 and T_M_ = 80.2°C) was collected from a door handle leading to a physician’s workroom in the Emergency Department (Table 3 and Figure 1(c)). Measurement of this sample was repeated (n = 3), revealing the presence of approximately 20 copies of viral RNA on this surface.

*Culture of VTM:* To determine if viable SARS-CoV-2 could be recovered from the two fomite samples that tested positive in the Spike assay, VTM from these samples was inoculated onto cultured Vero E6 monolayers. Despite the presence of CPE in wells inoculated with SARS-CoV-2 (positive biological control), no CPE formation was observed in wells inoculated with either the two positive fomite samples after 8 days in culture or media alone (negative biological control) (Table 4).

**Table 4.** Viral growth on Vero E6 cells.

Sample type	Viral growth on Vero E6 cells
Door threshold outside of patient’s room following HCP encounter	–
Door handle to physician workroom (Emergency Department)	–
SARS-CoV-2 USA WA1/2020 (positive control)	+
Media alone (negative control)	–

HCP: healthcare provider; SARS-CoV-2: severe acute respiratory syndrome coronavirus 2.

## Discussion

SARS-CoV-2 infected secretions expelled by patients while coughing, sneezing, or talking are known to transmit disease. Larger respiratory droplets (>5–10 µm) contaminated with the virus may also settle onto surfaces and result in indirect viral spread. A study at a shopping mall in Wenzhou, China, implicated fomites as a source of SARS-CoV-2 spread.^10^ In addition, viral shedding was detected in air and surface samples collected from COVID-19 patient rooms at the Nebraska Medical Center.^4^ When a subset of the samples was examined for viable virus using Vero E6 cells, two samples showed evidence of CPE, suggesting the presence of replicating virus. Another study in a hospital setting in Singapore found the presence of SARS-CoV-2 on numerous surfaces, including air vents, bed rails, electric switches, and toilet seats.^5^ However, the samples were not cultured to determine viral viability. Another investigation, which collected 26 samples from fomites in COVID-19 patient areas, identified the presence of SARS-CoV-2 RNA on two swabs from the plastic portion of CPAP helmets (located in proximity to the patient’s face), but failed to infect Vera E6 cells, indicating lack of viable virus.^11^ The present study also found evidence of SARS-CoV-2 RNA on high-touch surface areas outside of room of COVID-19 patients and at HCP workstations. However, viable virus was not recovered from the fomite samples. This finding is in agreement with other reports indicating that the spread of SARS-CoV-2 from fomites may be less extensive than originally suspected,^11–13^ despite the number of samples collected and the study period being limited in our investigation.

Although large inoculums of SARS-CoV-2 (10^4^ infectious viral particles) have been shown to survive on non-porous surfaces, such as glass and stainless steel for up to 72 h,^5^ it is unlikely that respiratory droplets would contain comparably high viral loads. In a recent study, approximately 1000 viral copies of coronavirus OC43 were detected in respiratory droplets collected over 30 min.^14^ However, the presence of a protein-rich medium, as found in airway secretions, could protect the expelled virus from environmental factors, such as temperature and humidity, and in turn, may enhance fomite persistence.^15^ Nonetheless, implementation of extensive and frequent cleaning of high-touch surfaces with viricidal cleaners in most hospital settings, such as UNMH, likely enhances viral inactivation and reduces transmission of the virus through fomites.

Since viral shedding of SARS-CoV-2 is typically highest during the earlier phases of the disease course, the study focused on HCP patient encounters during the first 3 days of hospitalization. To determine if aerosolized viral particles had the potential to contaminate HCP, samples were collected from the HCP before and after the patient encounters from exposed skin, as well as around the nose and mouth, and then tested for the presence of SARS-CoV-2 RNA. Viral RNA was not detected on either exposed skin or the nose and mouth (under the procedure mask/N95), regardless of direct contact time, and irrespective of whether the patient spoke, coughed, wore a surgical mask, or was using a nasal cannula. Since aerosolized SARS-CoV-2 viral particles are known to remain suspended in the air for minutes to hours, the COVID-19 isolation rooms were retrofitted with high flow HEPA exhaust fans to provide a minimum of 12 air changes per hour. This process likely minimized the amount of aerosolized viral particles circulating in the patient’s room and may have contributed, at least in part, to the absence of SARS-CoV-2 RNA detected on the exposed skin of the HCP. Although not directly evaluated in the current study, we propose that utilization of filtration units in the rooms of patients with SARS-CoV-2 is an important parameter for reducing spread of the virus in hospital settings. For example, several studies have found the presence of SARS-CoV-2 RNA fomites on multiple surfaces directly adjacent to and in close proximity to the patient.^4,10^ A limitation of the current study is the lack of sample collection directly within the patient’s room, thereby, not allowing us to determine if the use of filtration units impacted on the detection of positive skin flora and fomite samples.

Although the CDC RT-PCR diagnostic panel (N1 and N2) failed to detect any positive samples, the SYBR Green-based Spike qRT-PCR assay detected SARS-CoV-2 RNA on the door threshold outside a patient’s room following an encounter with HCP and on a door handle for a physician’s workroom. The C_T_ values of the two positive samples were approximately 31 and 38, which corresponded to ∼1000 and ∼20 viral particles, respectively. Since SYBR Green can bind to any amplified product (i.e. target or non-target), melting curve analysis (SYBR Green dye dissociation assays) were incorporated into the diagnostic platform to validate the specificity of the amplification. Specificity can be inferred as amplicons of a defined sequence that exhibit a single dissociation peak and melting temperature (T_M_) (Figure 1(a)). Since amplified products from two of the fomite samples had melting curves that overlapped that of the positive control (Figure 1(b) and (c)) and had melting temperatures within 1°C of the target amplicon, these samples were deemed positive. However, although these two samples indicate the presence of SARS-CoV-2 RNA on the respective surfaces, VTM from both samples failed to elicit viable virus upon culturing. These results may be explained by low levels of culturable virus in the samples, as indicated by the high C_T_ values (31 (∼1000 viral particles) and 38 (∼20 viral particles), respectively). It is important to point out that the fomite samples which tested positive using the molecular assay may have been viable at some point prior to sampling and, therefore, may have previously had the potential for transmission of the virus.

In conclusion, data presented here suggest that SARS-CoV-2 RNA was not widely disseminated in the specific locations sampled within the hospital environment. This may be explained, at least in part, by the fastidious use of virucidal cleaners on the high-touch surfaces sampled, along with the implementation of the high flow HEPA filtrations units. However, since the current study design does not directly address the impact of these preventative measures on SARS-CoV-2 detection and viability, we cannot definitively determine their consequences on the findings presented here. Although we did not detect viable virus from the small number of samples that were positive for the presence of SARS-CoV-2 RNA, this does not rule out the potential of viral transmission from fomites.

## Supplemental Material

sj-pdf-1-ebm-10.1177_15353702211024597 - Supplemental material for COVID-19 global pandemic planning: Presence of SARS-CoV-2 fomites in a university hospital setting Click here for additional data file. Supplemental material, sj-pdf-1-ebm-10.1177_15353702211024597 for COVID-19 global pandemic planning: Presence of SARS-CoV-2 fomites in a university hospital setting by Christopher Bartlett, Jens Langsjoen, Qiuying Cheng, Alexandra V Yingling, Myissa Weiss, Steven Bradfute, Douglas J Perkins and Ivy Hurwitz in Experimental Biology and Medicine
