# Supplementary material for: COVID-19 global pandemic planning: Presence of SARS-CoV-2 fomites in a university hospital setting
Source: Exp Biol Med (Maywood). 2021 Jul 4;246(18):2039–45. doi: 10.1177/15353702211024597 (PMC8461048; doi:10.1177/15353702211024597)
Supplement: sj-pdf-1-ebm-10.1177_15353702211024597 - Supplemental material for COVID-19 global pandemic planning: Presence of SARS-CoV-2 fomites in a university hospital setting [file sj-pdf-1-ebm-10.1177_15353702211024597.pdf]

SUPPLEMENTAL MATERIAL

Supplemental Questionnaire:

1. Did you come into physical contact with the patient?
2. Did the patient cough or sneeze?
3. Did the patient speak?
4. Was the patient wearing a nasal cannula or high flow nasal cannula?
5. Was the patient receiving a nebulization treatment?
6. Was the patient wearing a surgical mask?
7. Do you remember touching your head, neck, or other area of exposed skin?

Supplemental Figure:

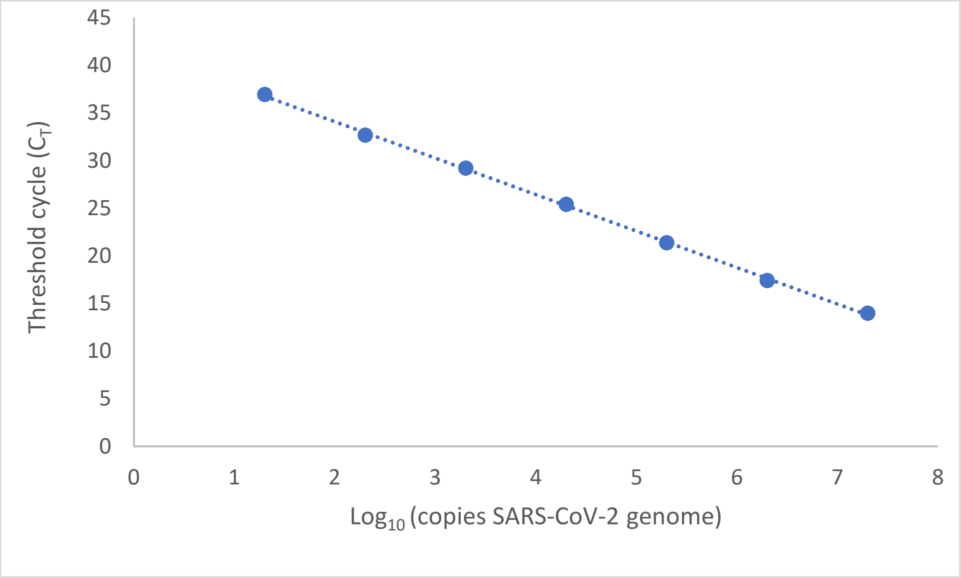

Supplemental Figure: Amplification of a 121 bp fragment of the *S*-gene from 2 to 2 x 10^7 copies of SARS-CoV RNA using the Spike RT-qPCR assay. The dynamic range of this assay is within 7 orders of magnitude, with a theoretical lower limit of detection (y-intercept) at C_T=41.76. The efficiency of this reaction is 82.45%.
